# Validity and reliability of the Brazilian version of the Illness Perception Questionnaire-Revised for Dental

**DOI:** 10.1590/1807-3107bor-2024.vol38.0029

**Published:** 2024-08-05

**Authors:** Marjorie Izabella Batista AGUIAR, Maria Beatriz Duarte GAVIÃO, Rogério Lacerda dos SANTOS, Renata Andréa Salvitti de Sá ROCHA, Taís de Souza BARBOSA

**Affiliations:** (a) Universidade Federal de Juiz de Fora – UFJF, Faculty of Dentistry, Juiz de Fora, MG, Brazil.; (b) Universidade Estadual de Campinas – Unicamp, Piracicaba Dental School, Department of Health Sciences and Pediatric Dentistry, Piracicaba, SP, Brasil,; (c) Universidade Federal de Juiz de Fora – UFJF, Department of Dentistry, Governador Governador Valadares, MG, Brasil.; (d) Universidade Federal de Campina Grande – UFCG, Faculty of Dentistry, Patos, PB, Brasil.

**Keywords:** Dental Caries, Parents, Surveys and Questionnaires, Validation Study

## Abstract

This study aimed to test the validity and reliability of the Brazilian version of the “Illness Perception Questionnaire-Revised for Dental” (IPQ-RD) in parents/guardians of children aged six to 14 years. The sample consisted of 63 parents/guardians of schoolchildren from the municipal school system of Teófilo Otoni, MG. Remote and virtual data collection consisted of self-completion of personal data, socioeconomic questionnaire and the Brazilian short versions of the “Parental-Caregiver Perceptions Questionnaire” (16-P-CPQ) and the “Family Impact Scale” (4-FIS). The IPQ-RD was applied by telephone interview. Almost half of the sample belonged to socioeconomic classes C1 and C2. Approximately 1/3 classified their child’s oral health as “regular” or “poor”, while 11.1% reported “strong” or “very strong” impact on their child’s well-being. The items most frequently cited as having an impact on the four domains of the 16-P-CPQ were: “bad breath” (23.8%), “mouth breathing” (20.7%), “feeling anxious or afraid” (20.7%), and “paying attention at school” (10%). In the 4-FIS, 11.1% “had little time for themselves or the family”. There were higher IPQ-RD scores in the “disease coherence” domain for women and lower values of “emotional dimensions” for parents/guardians with incomplete education. The mean IPQ-RD score was 126.4 (±15.1), and domain scores were positively correlated. The internal consistency was “almost perfect” for the IPQ-RD total score, ranging from “moderate” to “almost perfect” for the “child-control” and “child-consequences” domains. The intraclass correlation coefficient ranged from 0.04 (poor) to 0.68 (substantial). The Brazilian Portuguese version of the IPQ-RD proved to be valid and reliable for assessing the cognitive and emotional perception of parents/guardians about childhood dental caries.

## Introduction

Dental caries, a disease that affects about 54% of the Brazilian population, has several negative impacts on children’s lives, including early tooth loss and impaired quality of life for themselves and their families.^1^ The etiology of dental caries in children is directly associated with factors such as eating habits, irregular oral hygiene, and socioeconomic characteristics. Studies show a higher prevalence of dental caries in children from families with unfavorable socioeconomic conditions and that these parents/guardians have greater difficulty in perceiving the disease in their children.^1-3^

Social and economic circumstances influence behavior and perceptions of oral health of the individual; these factors, consequently, affect health outcomes,^1,4^ which makes it difficult for professionals to control complex health risk factors. The literature shows that parents/guardians with lower socioeconomic status have less knowledge of the factors associated with dental caries and oral hygiene, as well as less access to health services.^1^ Thus, the lack of knowledge about the oral health of the child and the efficacy of preventive strategies for caries disease is a barrier to promoting healthy oral hygiene practices in their children and to seeking dental care.^5,6^

Oral health-related quality of life (OHRQoL) is a multidimensional concept that addresses the individuals’ perception of the impact of their oral health on important factors in their daily lives, mainly related to social context and theories of cognitive and health behaviour.^7,8^ OHRQoL measurement tools evaluate the impact of oral health status on quality of life and psychosocial factors through the self-perception of individuals. For young children who have difficulty verbalizing emotions, measures of perception of parents/guardians, the primary caregivers of the child’s health, are used.^9^ Thus, measures to assess the perception of parents/guardians about the oral health of their children have been developed and their translated and cross-culturally adapted versions have been widely used in different countries and cultures.^9^

The process of translation and validation of a questionnaire requires a careful methodology composed of several stages, which result in adequate translation and coherent cross-cultural adaptation to the cultural context of the population in which the instrument will be used.^10,11^ Determining of the validity and reliability of the instrument is important because it allows the questionnaire to be used in another country, taking into account the cultural and social aspects of the new region. These processes must be performed effectively so that the inferences made from the data collected have no errors and the constructs evaluated in the translated questionnaire have the same accuracy as the original instrument.^12^

The “Illness Perception Questionnaire-Revised for Dental” (IPQ-RD) is a recently adapted for dentistry version of the “Illness Perception Questionnaire” (IPQ) originally developed in English to evaluate five cognitive domains (identity, causes, consequences, control, and course) of the disease.^13^ The revised version of the instrument (“Revised Illness Perception Questionnaire”) improved the theoretical foundations of the original version from the inclusion of two domains – disease coherence and emotional representations.^14^ In 2016, this version was adapted for dentistry as a complete and dynamic measure to understand how parents/guardians formulate the cognitive and emotional representations/perceptions of the child’s oral disease in response to the risk of disease of the children and the factors that determine their coping strategies.^8^ In a previous study, the original version of IPQ-RD was translated into Brazilian Portuguese and socioculturally adapted for use in the Brazilian parent/guardian population, a validated version of the instrument does not yet exists.^15^

Considering the importance of parents/guardians as the main caregivers of the child’s oral health, who must provide an accurate representation of caries disease for effective adherence to disease self-regulatory strategies,^16^ the present study aimed to validate and test the reliability of the Brazilian version of the IPQ-RD in parents/guardians, based on the association with personal and socioeconomic characteristics and the OHRQoL of children and their families.

## Methodology

### Study design

This cross-sectional and quantitative study was approved by the Ethics and Research Committee of the Federal University of Juiz de Fora under the CAAE protocol 16525219.0.0000.5147.

### Setting

This study was performed from March to December, 2021. The data were collected remotely and virtually through the WhatsApp^®^ and Google^®^ Forms apps. The link and a brief explanation about the survey was sent by the teachers to the parents’ WhatsApp group. Through the link, parents were able to access information about the survey, the informed consent form (In Google^®^ Forms format), and a question about whether or not they agreed to participate in the survey. Those who agreed to participate filled the form with personal information (full name of the responsible person, telephone number, best time for a telephone interview, and gender and age of the child) and completed the socioeconomic and OHRQoL questionnaires (Parental-Caregiver Perceptions Questionnaire – 16-P-CPQ and Family Impact Scale – 4-FIS).

The IPQ-RD was applied by telephone by a previously trained researcher. The training consisted of applying the IPQ-RD through telephone interviews with two parents/guardians, who did not participate in the final sample. The interview followed a pre-structured script and each phone call was timed. After the training, the estimated time for the application of the questionnaire and a proposal for a standardized final script for the application of the IPQ-RD in the total sample were established.

### Participants

The link to the form was sent to parents/guardians of children aged between six and fourteen years enrolled in 1st year in elementary school I through 9th year of elementary school II. Seven classes of each year were selected in the Municipal School Sister Maria Amália and two classes of each year were selected in the Municipal School Sidônio Ottoni. Both schools are located in the municipality of Teófilo Otoni, MG.

Teófilo Otoni is located in the interior of Minas Gerais and belongs to the mesoregion of the Mucuri Valley, located about 450 km northeast of the state capital. It occupies an area of 3,242,270 km^2^, and its population was estimated in 2021 at 141,269, making it the 17th most populous city in the state (IBGE, 2022). The Human Development Index (HDI) of Teófilo Otoni is 0.70 (IBGE, 2010), the Gross Domestic Product (GDP) per capita is R$ 19,191.15, 29.85% of its inhabitants live below the poverty line, and more than 22% of the population does not have access to adequate sanitation.^17^

The inclusion criteria were the signing of the informed consent and the completion of personal data form and questionnaires. Parents/guardians who did not agree to participate in the research and who did not provide essential personal data, such as telephone number, were excluded. Finally, 67 parents/guardians answered the questionnaire, 4 of which were excluded for not providing their phone number. In total, the convenience sample consisted of 63 parents/guardians, of which thirteen answered the IPQ-RD a second time after 1 month to test the reproducibility of the instrument.

The sample size was calculated based on the Brazilian version of the IPQ-RD total score obtained from a pre-test study with 15 Brazilian parents/caregivers of pediatric patients.^15^ Considering a mean total score of 124.1, a standard deviation of 19.6, a sampling error of 5%, a confidence level of 95%, and a correction factor of 1.234, the required sample size was defined as 47 parents/caregivers.

### Variables

#### Outcome variable

The Brazilian Portuguese version of the IPQ-RD,^15^ originally developed in English,^8^ was used to evaluate the emotional and cognitive/representative perception of parents/guardians about dental caries in children. The instrument consists of 33 multiple-choice questions and an open-ended question (item 34) divided into cognitive dimensions and emotional representation (Table 1). The response options for each item are given on a 5-point Likert scale ranging from “totally agree” (score 1) to “totally disagree” (score 5). The score of each domain depends on the number of items and the total score is the sum of domain scores; the higher the score, the lower the perception of the disease.

**Table 1:** Domains, number of items and variation of IPQ-RD score.

Domains	Definitionn	No. Items	Variation of the score
Cognitive dimensions			
1 – Identity*	Parents/guardians' perception of the intensity of symptoms associated with dental caries in the child	2	2/10
2 – Child consequences*	Beliefs about the deleterious consequences of dental caries on the child's daily activities	7	7/35
3 – Parent/parent consequences*	Beliefs about the deleterious consequences of the child's dental caries in their own daily activities	5	5/25
4 – Child-control*	Beliefs whether dental caries and symptoms can be prevented, improved or kept under control by the child's actions	4	4/20
5 – Parental/Responsible Control*	Beliefs whether dental caries in the child and its symptoms can be prevented, improved or kept under control by their own actions	4	4/20
6 – Chronic-course*	Beliefs about the chronicity and duration of dental caries in the child	2	2/10
7 – Cyclic course*	Beliefs about predictability and symptoms of dental caries in children	2	2/10
8 – Disease coherence*	If parents/guardians have a clear understanding of dental caries and symptoms in the child	2	2/10
Emotional dimensions**	Evaluation of the emotional response of parents/guardians to the presence of dental caries in the child	4	4/20
Causes of the disease	Perception of the causes of dental caries in children	1 (11 subitems)	11/55
Causes of the disease (open question)	Three most important causes of the disease	1	–

#### Independent variables

To evaluate socioeconomic data, the instrument “Brazil Economic Classification Criterion”^18^ was used, which categorizes families into economic classes based on the level of education of the head of the family and the number of consumer goods reported by parents/guardians, such as washing machine, freezer, DVD player, personal computer, dishwasher, microwave oven, refrigerators, motorcycles, and automobiles. Scores were assigned to each item and the total sum was used to classify families in Class A (45 to 100 points), Class B1 (38 to 44 points), Class B2 (29 to 37 points), Class C1 (23 to 28 points), Class C2 (17 to 22 points), and Class D/E (0 to 16 points).

The Brazilian short version of the P-CPQ with 16 items^19,20^ was used to evaluate the perception of parents/guardians about the impact of oral diseases on the quality of life of children. The 16-P-CPQ questions refer to oral symptoms, functional limitations, emotional well-being, and social well-being domains, each with four items, with response options ranging from “never” (score 0) to “every day or almost every day” (score 4). The response “I don’t know” was scored 0 because in most cases children would answer “never” if the parent/guardian didn’t know the answer to an item. There are also two questions about the overall perception of parents/guardians about the oral health and general well-being of the child, with response options ranging from zero (0) to four (4) points. The total score was obtained by the sum of the scores of all questions and, for each domain, the sum of the scores of the specific items. The higher the score, the greater the impact of oral diseases on the child’s quality of life.

The impact of oral diseases on family functioning was evaluated by the Brazilian short version of the FIS (4-FIS).^22^ The four items have as answer options: ‘Never’ (score 0), ‘Once or twice’ (1), ‘Sometimes’ (2), ‘Often’ (3), and ‘Every day’ or ‘Almost every day’(4). The answer “I don’t know” was assigned the score 0. The total score of the 4-FIS was obtained by the sum of the scores of the four items, ranging from 0 to 20 points; the higher the score, the greater the impact on family functioning.

## Statistical analysis

Statistical analysis was performed with the Statistical Package for Social Sciences (SPSS) 23.0 (IBM Corp. released 2015. IBM SPSS Statistics for Windows, Version 23.0. Armonk, USA) and BioEstat. 5.3 (Mamirauá Institute for Sustainable Development, Belém, Brazil). The significance level considered was α=0.05. The chi-square partition/independence test was used to evaluate the differences in the distribution of independent variables (personal, socioeconomic and oral health-related quality of life data– 16-P-CPQ and 4-FIS).

Criterion validity was tested by comparing the IPQ-RD scores between the categories of independent variables (gender, age, educational level, social class, responses of global perception of oral health and general well-being of 16-P-CPQ) by the Mann-Whitney or Kruskal-Wallis tests (Student’s t-test), where appropriate. Spearman’s correlation test was used to evaluate construct validity by the association between IPQ-RD scores with 16-P-CPQ and 4-FIS scores.

The internal consistency of the instrument was evaluated by the association between the IPQ-RD (Spearman correlation test) domains and by calculating Cronbach’s alpha coefficient and alpha if the domain is excluded. Coefficient values above 0.80 represent “near-perfect” internal consistency, 0.61 to 0.80, “substantial”, 0.41 to 0.60, “moderate”, 0.21 to 0.40, “poor”, and ≤ 0.21 “weak”.^23^

The test-retest reliability of the instrument was evaluated by reapplying the IPQ-RD in 20% of the sample (n = 13) randomly selected after approximately 1 month for the calculation of the intraclass correlation coefficient (ICC). ICC values between 0.81 and 1.0 represent “near-perfect” test-retest reliability, 0.61 to 0.80, “substantial”, 0.41 to 0.60, “moderate”, and <0.40, “poor”.^24^

## Results

Table 2 shows the personal and sociodemographic characteristics of the participants. Almost all respondents were mothers (92.1%), 44.4% had a car, 55.6% did not have a monthly domestic employee, 71.4% had a washing machine, 73% had a bathroom, 65.1% did not have a DVD player, 95.2% had a refrigerator, 63.5% did not have a freezer, 44.4% had a microcomputer, and 96.8% did not have a dryer. The main source of water was the general distribution network, reported by 92.1% of the sample, and 79.4% lived on a paved street. No participant had a dishwasher.

**Table 2:** Personal and sociodemographic characteristics of the study participants (n = 63).

Features	n	%	p-value
Personal data			
Responder			
Mother	58	92.1	< 0.0001*
Father	4	6.3	
Grandmother	1	1.6	
Education level			
Incomplete Fundamental I	6	9.5	0.099*
Incomplete Fundamental II	9	14.3	
Incomplete middle school	12	19.0	
Incomplete university	17	27.0	
Complete university	19	30.2	
Child sex			
Female	36	57.1	0.439**
Male	27	42.9	
Child’s age (years)			
6-7 years	12	19.0	0.098*
8-10 years	25	39.7	
11-14 years	26	41.3	
Sociodemographic data			
Number of passenger cars			
1	28	44.4	< 0.0001*
2	7	11.1	
3	4	6.3	
None	24	38.1	
Number of monthly domestic employees (5x week)			
1	21	33.3	< 0.0001*
2	6	9.5	
3	1	1.6	
Does not have	35	55.6	
No washing machines (except washing sink)			
1	45	71.4	< 0.0001*
2	4	6.3	
Does not have	14	22.2	
Number of bathrooms			
1	46	73.0	< 0.0001*
2	13	20.6	
3	3	4.8	
Does not have	1	1.6	
Number of DVD players			
1	18	28.6	< 0.0001**
2	3	4.8	
3	1	1.6	
Does not have	41	65.1	
Number of refrigerators			
1	60	95.2	< 0.0001**
2	3	4.8	
Number of freezers			
1	22	34.9	< 0.0001*
2	1	1.6	
Does not have	40	63.5	
Number of microcomputers			
1	28	44.4	< 0.0001**
2	13	20.6	
3	1	1.6	
4 or more	2	3.2	
Does not have	19	30.2	
Number of dishwasher			
Does not have	63	100.0	-
Number of microwave			
1	36	57.1	0.439**
Does not have	27	42.9	
Number of dryers			
1	1	1.6	< 0.0001**
2	1	1.6	
Does not have	61	96.8	
Household water			
General distribution network	58	92.1	< 0.0001*
Well or mineral spring	4	6.3	
Both	1	1.6	
House street			
Asphalted/Paved	50	79.4	0.0002*
Earth/Gravel	13	20.6	

*Chi-square partition test. ^†^Chi-square independence test.

In relation to the socioeconomic classification of the population according to the Brazil Economic Classification Criterion, almost half of the sample belonged to class C1 (24%) or C2 (25%), 13% and 19% belonged to B1 and B2, respectively, 5% to class A, and 14% to classes D and E.

Table 3 shows the distribution of responses for the 16-P-CPQ and 4-FIS scales. The 16-P-CPQ results showed that the oral health of the child was considered “regular” or “bad” by 28.6% and 6.3%, whereas the magnitude of the impact of oral health on the child’s overall well-being was classified as “high” and “very high” in 6.3% and 4.8% of the sample.

**Table 3:** Distribution [n (%)] of the answer options (score) for the scales 16-P-CPQ and 4-FIS (n = 63).

1. How would you rate the health of your child’s teeth, lips, jaws, and mouth?	Excellent (score 0)	Very good (score 0)	Good (score 2)	Regular (score 3)	Bad (score 4)	p-value
	4 (6.3)	14 (22.2)	23 (36.5)	18 (28.6)	4 (6.3)	
2. How much is your child’s overall well-being affected by the condition of their teeth, lips, jaws or mouth?	**Not at all (score 0)**	**Only a little (score 1)**	**More or less (score 2)**	**Very much (score 3)**	**Very much (score 4**	
	19 (30.2)	21 (33.3)	16 (25.4)	4 (6.3)	3 (4.8)	p = 0.0005
**Oral**	**Never (score 0)**	**Once or twice (score 1)**	**Sometimes (score 2)**	**Frequently (score 3)**	**Every day or almost every day (scoe 4)**	
3. Has your child had pain in his/her teeth, lips, jaws or mouth?	24 (38.1)	19 (30.2)	18 (28.6)	2 (3.2)	0 (0.0)	p < 0.0001
**Symptoms**						
4. Has your child had mouth injuries?	31 (49.2)	19 (30.2)	13 (20.6)	0 (0.0)	(0.0)	p < 0.0001
5. Has your child had bad breath?	17 (27.0)	11 (17.5)	21 (33.3)	11 (17.5)	4 (6.3)	p = 0.0001
6. Has your child had food trapped inside or between his or her teeth?	10 (15.9)	11 (17.5)	29 (46.0)	9 (14.3)	4 (6.3)	p = 0.0002
**Functional limitations**						
7. Has your child had difficulty biting or chewing foods such as apple, corn cob, or though meat?	38 (60.3)	6 (9.5)	10 (15.9)	7 (11.1)	(3.2)	p < 0.0001
8. Has your child had mouth breathing?	21	6 (9.5)	23 (36.5)	11 (17.5)	2(3.2)	p = 0.0002
9. Has your child had problems during sleep?	35 (55.6)	14 (22.2)	11 (17.5)	3 (4.8)	0 (0.0)	p < 0.0001
10. Has your child had difficulty drinking or eating hot or cold foods?	32 (50.8)	9 (14.3)	14 (22.2)	7 (11.1)	1 v	p < 0.0001
**Emotional welfare**						
11. Does your child feel angry or frustrated?	30 (47.6)	8 (12.7)	20 (31.7)	2 (3.2)	3 (4.8)	p < 0.0001
12. Does your child feel anxious or afraid?	18 (28.6)	6 (9.5)	26 (41.3)	10 (15.9)	3 (4.8)	p < 0.0001
13. Does your child act timidly or with shame?	20 (31.7)	8 (12.7)	28 (44.4)	5 (7.9)	2 (3.2)	p < 0.0001
14. Has your child worried about what other people think about their teeth, lips, mouth, or jaws?	34 (54.0)	4 (6.3)	15 (23.8)	5 (7.9)	5 (7.9)	p<0.0001
**Welfare**						
15. Has your child missed school (e.g. pain, consultations, surgeries)?	36 (57.1)	13 (20.6)	14 (22.2)	0 (0.0)	0 (0.0)	p < 0.0001
16. Has your child had difficulty paying attention to school?	33 (52.4)	6 (9.5)	18 (28.6)	6 (9.5)	0 (0.0)	p < 0.0001
17. Has your child avoided smiling or laughing when he is around other children?	42 (66.7)	4 (6.3)	11 (17.5)	4 (6.3)	2 (3.2)	p < 0.0001
18. Has your child been teased or nicknamed by other children?	43 (68.3)	6 (9.5)	11 (17.5)	1 (1.6)	2 (3.2)	p < 0.0001
**Short version - 4-FIS**						
1. Have you or another family member felt disturbed?	22 (34.9)	10 (15.9)	29 (46.0)	0 (0.0)	2 (3.2)	p < 0.0001
2. Have you or another family member had interrupted sleep?	24 (38.1)	5 (7.9)	31 (49.2)	0 (0.0)	3 (4.8)	p < 0.0001
3. Have you or another family member had little time for yourself or the family?	18 (28.6)	17 (27.0)	36 (57.1)	0 (0.0)	7 (11.1)	p < 0.0001
4. Has your child become jealous of you or another family member?	26 (41.3)	7 (11.1)	26 (41.3)	0 (0.0)	4 (6.3)	p < 0.0001

16-P-CPQ: parental-caregiver perceptions questionnaire; 4-FIS: family impact scale. p-value obtained by the Chi-square partition test.

Regarding oral symptoms, 3.2% reported that their child had toothache “frequently”; 23.8% had bad breath “often/every day or almost every day”; 20.6% had food trapped inside or between their teeth “often/every day or almost every day”. Approximately 14% reported difficulty biting and chewing hard foods, “often/every day or almost every day”; 20.7% had mouth breathing; 4.8% had sleep problems “frequently”; 12.7% had difficulty drinking or eating hot or cold foods “often/every day or almost every day”. In the emotional well-being domain, 8% reported that their child feels irritated or frustrated “often/every day or almost every day”; 20.7% that the child feels anxious or afraid; 11.1% that the child acts timidly or with shame; 15.8% that the child worries about what other people think about their teeth, lips, mouth or jaws. Approximately 10% of the sample reported that the child “frequently” had difficulty paying attention at school; 9.5% that the child avoided smiling or laughing when they were around other children “often/every day or almost every day”; 4.8% that the child was provoked or nicknamed by other children.

On the 4-FIS scale, 3.2% reported that “every day or almost every day” he or she or another family member felt disturbed; 4.8% had interrupted sleep; 11.1% had little time for themselves (a) or the family; 6.3% that the child was jealous of him/her or other family members.

Table 4 shows the criterion validity of the IPQ-RD, with women showing a higher mean for the domain “disease coherence” than men (5.0 vs. 4.3; p = 0.034). There was a significant difference in the scores of the domain “emotional dimensions”, with higher scores for parents/guardians with complete fundamental level I and II compared to those with incomplete middle education. Those with incomplete higher education had higher scores than those with incomplete middle education (p = 0.011). There was no significant difference in the IPQ-RD scores between the age and social class categories.

**Table 4:** Criteria validity: mean (± SD) of the IPQ-RD scores according to the categories of independent variables (n = 63).

Independent variables	n	Identity	Consequences		Control		Course		Coherence of the disease	Dimensions Emotional	Causes of sickness	Score total	p-value

			Child	Parents/guardians	Child	Parents/guardians	Chronic	Cyclic					

		2–10	7–35	5–25	4–20	4–20	2–10	2–10	2–10	4–20	11–55	43–215	
Sex^*^													p = 0.034
Female	36	4.8 (1.9)	22.7 (5.6)	16.9 (3.6)	8.9 (2.2)	8.9 (2.2)	5.9 (1.4)	5.3 (1.8)	5.0 (1.6)	10.7 (2.7)	37.6 (5.0)	126.9 (14.8)	
Male	27	4.4 (1.6)	22.0 (6.3)	16.3 (3.7)	8.3 (2.4)	9.2 (2.5)	5.9 (1.8)	4.8 (1.5)	4.3 (1.4)	11.2 (3.3)	36.7 (7.2)	122.7 (24.4)	
Age (years)^**^													
6–7	12	4.3 (1.6)	21.5 (6.2)	15.3 (3.8)	8.2 (2.4)	8.8 (2.4)	6.0 (2.0)	4.6 (1.5)	4.3 (1.6)	10.8 (3.3)	36.4 (7.1)	119.8 (23.1)	
8–10	25	4.6 (1.6)	22.3 (6.1)	16.3 (3.8)	8.3 (2.4)	8.7 (2.5)	5.8 (1.6)	4.8 (1.7)	4.7 (1.6)	10.7 (3.1)	37.1 (6.9)	122.8 (22.8)	
11–14	26	4.3 (1.8)	22.2 (5.8)	16.6 (3.9)	8.8 (2.5)	9.5 (2.4)	5.8 (1.8)	5.5 (1.8)	4.7 (1.5)	11.2 (3.1)	36.3 (7.2)	124.5 (22.9)	
Education level^**^													p = 0.018
Incomplete Fundamental I	6	4.0 (2.2)	20.7 (4.7)	16.7 (2.2)	7.7 (1.9)	9.3 (2.7)	6.7 (1.0)	5.3 (2.4)	4.8 (1.8)	12.5 (2.7)^a^	38.7 (5.8)	126.3 (16.5)	
Incomplete Fundamental II	9	4.9 (1.8)	22.9 (5.5)	17.1 (3.8)	8.3 (1.9)	9.4 (1.9)	5.7 (1.6)	5.1 (1.5)	4.0 (1.0)	12.6 (2.4)^a^	38.7 (4.2)	128.7 (15.5)	
Incomplete middle	12	5.5 (1.9)	22.7 (6.2)	16.3 (3.8)	9.5 (2.5)	8.1 (1.9)	5.8 (1.7)	4.7 (1.6)	5.5 (2.2)	9.1 (2.2)^b^	36.7 (4.4)	123.8 (17.3)	
Incomplete university	17	4.2 (1.8)	22.2 (6.6)	17.0 (3.6)	8.5 (2.3)	9.6 (2.4)	6.3 (1.8)	4.9 (1.6)	4.3 (1.3)	11.5 (3.3) ^AC^	37.9 (4.2)	126.5 (13.4)	
Complete university	19	4.3 (1.2)	23.6 (4.8)	16.7 (3.3)	8.7 (2.3)	9.4 (2.3)	5.7 (1.6)	5.5 (1.7)	4.7 (0.9)	10.8 (2.8)^abc^	37.6 (5.8)	126.9 (15.9)	
Social class^**^ (points)													
A (45–100 )	3	5.0 (3.0)	23.3 (8.1)	17.0 (3.6)	9.7 (2.5)	12.3 (0.6)	7.3 (1.2)	4.0 (0.0)	5.3 (1.2)	13.0 (5.2)	42.3 (1.5)	139.3 (23.1)	
B1 (38–44)	8	3.8 (0.7)	21.4 (3.5)	16.4 (4.3)	8.4 (1.1)	9.0 (1.4)	5.0 (1.1)	5.0 (1.9)	4.1 (0.4)	10.5 (2.8)	34.5 (4.0)	118.0 (11.2)	
B2 (29–37)	12	4.6 (1.2)	22.4 (7.1)	15.9 (2.9)	8.9 (2.7)	9.6 (2.5)	6.0 (1.9)	5.4 (1.7)	4.5 (0.9)	10.6 (3.1)	38.2 (5.9)	126.1 (16.9)	
C1 (23–28)	15	4.4 (1.6)	23.8 (4.6)	17.2 (3.7)	8.9 (2.9)	8.7 (2.8)	6.0 (1.5)	5.4 (1.5)	4.4 (1.5)	11.9 (2.1)	38.3 (4.7)	128.9 (13.0)	
C2 (17–22)	16	4.7 (2.0)	20.8 (5.8)	15.9 (3.1)	8.4 (1.8)	9.0 (2.1)	6.1 (1.8)	4.8 (1.5)	5.5 (2.1)	9.9 (3.1)	37.1 (4.6)	122.2 (15.1)	
D-E (0–16)	9	5.1 (2.3)	25.6 (5.1)	18.9 (2.6)	8.2 (2.2)	8.9 (1.4)	6.0 (1.4)	5.3 (2.2)	4.0 (0.5)	12.3 (2.5)	39.0 (4.1)	133.3 (12.9)	
Global perception of oral health (16-P-CPQ)**													
Excellent/Very good	18	4.2 (1.5)	22.6 (4.9)	16.2 (3.8)	9.2 (1.8)	9.1 (1.7)	5.3 (1.4)	5.1 (1.7)	4.3 (0.8)	11.0 (2.5)	35.8 (5.0)	122.9 (14.3)	
Good	23	4.7 (1.9)	24.3 (4.9)	17.3 (3.3)	8.0 (2.4)	9.9 (2.6)	6.4 (1.6)	5.6 (1.7)	5.0 (1.6)	12.0 (3.0)	38.7 (4.9)	131.9 (13.5)	
Regular/Bad	22	4.7 (1.8)	21.0 (6.4)	16.6 (3.0)	8.9 (2.3)	8.5 (2.1)	6.0 (1.6)	4.6 (1.5)	4.6 (1.8)	10.2 (2.9)	38.3 (4.3)	123.5 (16.3)	
Global impact on general well-being (16-P-CPQ)**													
Not at all	40	4.5 (1.7)	22.8 (5.5)	17.1 (3.4)	8.4 (2.2)	9.4 (2.3)	5.9 (1.6)	5.3 (1.7)	4.5 (1.4)	11.3 (3.0)	38.2 (5.1)	127.1 (15.4)	
Just a little													
More or less	16	4.6 (1.9)	232 (5.6)	16.8 (2.9)	9.0 (2.1)	8.8 (2.0)	6.5 (1.3)	5.0 (1.5)	5.1 (1.6)	11.3 (2.5)	38.1 (3.9)	128.3 (11.9)	
Very/Very	7	4.9 (1.6)	20.9 (6.3)	15.1 (4.0)	9.6 (2.9)	9.1 (2.5)	5.4 (2.1)	4.6 (1.8)	4.6 (1.8)	9.4 (3.0)	34.4 (4.5)	118.0 (18.9)	

IPQ-RD: illness perception questionnaire-revised for dental; P-CPQ: parental-caregiver perceptions questionnaire.

[ ] : possible variation of the score. * Mann-Whitney test; ** Kruskal-Wallis test (Student’s t ).

Table 5 shows the results of construct validity. There was a significant negative correlation between the “identity” domain of the IPQ-RD and the “emotional well-being” domain of 16-P-CPQ (r = -0.31; p = 0.013). The domain “parental control/guardians” was also negatively associated with the domain “oral symptoms” of the 16-P-CPQ (r = -0.27; p = 0.032). The other domains were not significantly associated with the 4-FIS scale or with the 16-P-CPQ scale.

**Table 5:** Construct validity: correlation between P-CPQ and FIS scores with IPQ-RD (n = 63).

Variables	Total		16-P-CPQ								4-FIS	

			OS		LF		BEE		BES			

	n	p-value	n	p-value	n	p-value	n	p-value	n	p-value	n	p-value
Total score	0.07	0.575	0.04	0.752	0.08	0.509	0.09	0.492	0.00	0.978	0.06	0.661
Domains												
Identity	0.01	0.964	0.05	0.692	0.12	0.360	0.11	0.386	-0.31	0.013	0.00	0.973
Child consequences	0.05	0.712	0.13	0.298	0.04	0.764	0.03	0.793	-0.04	0.731	0.10	0.427
Consequences parents/guardians	0.11	0.393	0.09	0.477	0.10	0.447	0.11	0.400	0.05	0.706	0.06	0.632
Control-child	0.07	0.07	0.15	0.245	0.12	0.369	0.01	0.926	-0.03	0.794	0.11	0.389
Control parents/guardians	-0.07	0.566	-0.27		-0.04	0.854	-0.02		0.07		-0.16	
Course-chronic	-0.02	0.852	0.02		0.05	0.719	-0.09	0.464	-0.03	0.820	-0.01	0.951
Cyclic course	-0.05	0.717	-0.04	0.767	-0.05	0.689	-0.06	0.616	0.01	0.908	-0.07	0.599
Disease coherence	-0.03	0.828	-0.07	0.584	0.03	0.791	0.01	0.940	-0.09	0.506	0.03	0.809
Emotional dimensions	0.04	0.728	-0.12	0.338	0.03	0.798	0.07	0.583	0.14	0.273	0.01	0.916
Causes of the disease	0.10	0.437	0.05	0.700	0.05	0.699	0.13	0.292	0.07	0.594	0.05	0.708

IPQ-RD: illness perception questionnaire-revised for dental; P-CPQ: parental-caregiver perceptions questionnaire; SO: oral symptoms; LF: functional limitations; BEE: emotional well-being; BES: social welfare; FIS: family impact scale. r: Spearman correlation coefficient

The Brazilian version of the IPQ-RD demonstrated good internal consistency, with a significant correlation among the scores of the domains (p < 0.05), except for the domains “child-control”, “course-cyclic” and “disease coherence” that were not associated with any other domain (Table 6).

**Table 6:** Internal consistency of the IPQ-RD: correlation matrix between domains (n = 63).

Variables	Identity		Consequences				Control				Course				Coherence of the disease		Dimensions emotional	

			Child		Parents/guardians		Child		Parental responsible		Chronic		Cyclic					

	r	p-value	r	p-value	r	p-value	r	p-value	r	p-value	r	p-value	r	p-value	r	p-value	r	p-value
Consequences																		
Child	0.33	0.009	-															
Parents/guardians	0.25	0.047	0.69	< 0.0001	-													
Control																		
Child	0.19	0.139	0.16	0.221	0.14	0.291	-											
Parental control/Responsible	-0.07	0.566	0.03	0.795	0.11	0.393	0.00	0.984	-									
Course																		
Chronic	0.02	0.887	0.23	0.074	0.18	0.149	-0.06	0.653	0.21	0.104	-							
Cyclic	0.01	0.961	0.19	0.134	0.13	0.324	0.02	0.881	0.01	0.928	0.08	0.530						
Disease coherence	0.06	0.652	-0.06	0.617	-0.15	0.240	0.16	0.219	0.02	0.904	0.22	0.081	0.07	0.561	-			
Dimensions emotional	-0.04	0.751	0.39	0.002	0.35	0.006	0.09	0.492	0.37	0.003	0.23	0.065	0.10	0.447	-0.01	0.925	-	
Causes of the disease	0.19	0.127	0.30	0.018	0.49	< 0.0001	-0.16	0.213	0.12	0.368	0.29	0.021	-0.02	0.847	0.09	0.506	0.30	0.016

IPQ-RD: illness perception questionnaire-revised for dental. r: Spearman correlation coefficient.

Table 7 shows the reliability data of the questionnaire. The mean total IPQ-RD was 126.4 (± 15.1), the values ranged from 4.6 (± 1.7) for the domain “identity” to 37.8 (± 4.8) for “causes of the disease”. The internal consistency for the IPQ-RD total score was “almost perfect” (α = 0.85), and ranged from “moderate” (α = 0.43) to “almost perfect” (α = 0.89) for the domains “child control” and “child-consequences”, respectively. The ICC ranged from 0.04 to 0.68 indicating temporal stability range from “poor” to “substantial”.

**Table 7:** IPQ-RD reliability: floor and ceiling effects, Cronbach’s alpha coefficient for total scale, Alpha if domain is excluded (n=63) and reproducibility (n = 13).

Variables	No. items	Possible variation of the Score	Variation of score Found	Tread effect*/Ceiling effect^†^	Average (SD)	Internal consistency		Reproducibility

						Cronbach alpha	Alpha if domain is deleted	JRC (95%IC)
Cognitive dimensions								
Identity	2	1–10	2–9	0 (0.0)	4.6 (1.7)	0.82	0.84	0.42 (0.04–0.69)
Consequences								
Child	7	1–35	7–34	0 (0.0)	22 .7 (5.6)	0.89	0.77	0.45 (0.07–0.71)
Parents/guardians	5	1–25	10–24	0 (0.0)	16.8 (3.3)	0.74	0.81	0.24 (–0.15–0.58)
Control								
Child	4	1–20	4–15	0 (0.0)	8.7 (2.2)	0.43	0.86	0.26 (–0.13–0.59)
Parental control/Responsible	4	1–20	4–14	0 (0.0)	9.2 (2.2)	0.48	0.85	0.32 (–0.06–0.63)
Course								
Chronic	2	1–10	3–8	0 (0.0)	6.0 (1.6)	0.49	0.84	0.68 (0.40–0.84)
Cyclic	2	1–10	2–8	0 (0.0)	5.1 (1.7)	0.68	0.85	0.64 (0.34–0.82)
Coherence of the disease	2	1–10	2–8	0 (0.0)	4.7 (1.5)	0.68	0.85	0.42 (0.05–0.70)
Emotional dimensions	4	1–10	4–16	0 (0,0)	11.1 (2.9)	0.69	0.83	0.24 (–0.15–0.58)
Causes of the disease	1 (11 sub items)	1–55	29–47	0 (0,0)	37.8 (4.8)	0.69	0.83	0.04 (–0.35–0.42)
Total score	33	33–165	93–156	0 (0,0)	126.4 (15.1)	0.85	–	–

IPQ–RD: illness perception questionnaire–revised for dental; DP: standard deviation; ICC: intraclass correlation coefficient; CI: confidence interval; * % of respondents with score = 0; ^†^% of respondents with maximum score

## Discussion

Oral diseases, such as dental caries, can directly influence the quality of life of children and their families, since inadequate perception of parents/guardians about the disease can delay seeking dental treatment.^25^ In recent years, there has been an increase in studies evaluating the relationship between oral health conditions and quality of life, especially in children.^26^ Oral health instruments that measure oral health perception and correlation with well–being are extremely important for planning strategies of oral health prevention and care.^27^

This study tested the validity and reliability of the Brazilian version of IPQ–RD^15^ as a tool to investigate the perception/representation of parents/guardians regarding dental caries in children.^8^ This questionnaire is one of the first to be developed based on the *Common–Sense Model of Self–Regulation,* which understands the behavior of the individual towards disease from their perception of the representative, emotional, and cognitive aspects of disease.^28^ Understanding this process is a primary concern for health organizations in developing care models aimed at health promotion and disease prevention.^28,29^

Criterion validity establishes the validity of a measurement instrument by comparing it with external criteria. In this study, the IPQ–RD scores in the different categories of sex, age, educational level, social class, and overall perception of oral health and general well–being were compared. On average, higher scores were observed in females for the domain “disease coherence”, which determines whether parents/guardians have a clear understanding of dental caries and symptoms in the child. This result is explained by the fact that the majority of the sample (63%) consisted of people with lower socioeconomic status (especially class C, 49%),^18^ corroborating previous studies in Brazil that suggest that the lower the economic level, the lower the knowledge about oral hygiene habits.^30,31^ On the other hand, studies performed in other countries found that, in general, women have greater knowledge and perception about the oral health of their children than men.^32,33^

In the domain “emotional dimensions”, which assesses the emotional response of parents/guardians to dental caries in their child, lower IPQ–RD mean scores were found for those with incomplete high school compared to parents/guardians with lower (elementary I and II) and higher level of education (incomplete higher education). In the study of Padilla–Moledo *et al*.,^34^ parents with a complete higher education presented fewer complaints and consequently fewer concerns about their children’s oral health, considering that they have more confidence in their children (a) and believe that their children perform effective oral hygiene, which was also observed by Pohjola *et al*.^35^

In the present study, more than 1/3 of the parents/guardians classified the oral health of their child as “regular/bad”, while 11.1% reported “high/very high” impact of oral health on the overall well–being of the child. Similar data were found in studies conducted by Silva *et al*.^36^ and Corrêa–*Faria et al*.,^1^ which found a significant association between low socioeconomic levels and increased prevalence of dental caries due to lower commitment to oral hygiene of people with lower socioeconomic status.

The items most frequently reported in the 16–P–CPQ domains were “bad breath” (23.8%; oral symptoms); “mouth breathing” (20.7%; functional limitations), “feeling anxious or afraid” (20.7%; emotional well–being), “difficulty paying attention at school” (10%; social well–being). In the 4–FIS, 11.1% “had little time for themselves (a) or the family”. Halber *et al*.,^37^ reported that the difficulty of parents in managing their daily activities results in less time to take their children to the dentist, even when public oral health services are offered. These findings corroborate validation studies of P–CPQ and FIS conducted in Brazil,^20,22^ which demonstrated that the daily routine of parents/guardians have a great impact on their children’s oral health, since the oral conditions of children affect family activities and harm the emotional well–being of parents, which consequently generates family conflicts.

Construct validity refers to the degree to which an instrument consistently relates to other similar measurements derived from the same theory and concepts. For the construct validity of this instrument, quality of life criteria were established and tested by comparing the results with those of the 16–P–CPQ questionnaire and the 4–FIS scale. The domains “identity” (which refers to the perception of parents/guardians of the intensity of symptoms associated with their child’s dental caries) and “parental/guardian control” (which refers to believing that dental caries in their child and its symptoms can be prevented, improved or kept under control by their own actions) were negatively associated with the domains “emotional well–being” and “oral symptoms” of the 16–P–CPQ, respectively. These results confirm the relationship between the constructs of cognitive perception of caries disease and the perception of OHRQoL. The lower the perception of the intensity of symptoms related to caries disease, the better the perception of the emotional aspects of the child’s OHRQoL. The less the parent/guardian believes that dental caries in the child and its symptoms can be prevented, improved or kept under control by their own actions, the lower the frequency of oral symptoms impact.

To evaluate homogeneity, correlations between the scores of the domains were calculated. The greater the relationship between the domains, the greater their homogeneity. Moreover, the domains that are not correlated with the others (very low correlation coefficients, i.e., less than 0.2) should be eliminated to increase homogeneity. The Brazilian version of the IPQ–RD showed good homogeneity, with a significant correlation between the scores of the domains, except for three that were not associated with any other: “child control”, “cyclic course”, and “disease coherence”. These findings might be due to a probable lack of knowledge of parents regarding symptoms and characteristics of dental caries, considering that all domains that were not associated refer to their understanding of dental caries. Similar studies were conducted to assess parents’ perception of dental caries. Hooley *et al*.^38^ and Nelson *et al*.^8^ reported that parents/guardians perceive dental caries and seek dental care for their children only when pain is present or tooth loss is visible. Slusar & Nelson^5^ suggest that dentists must attempt to change this parental perception of dental caries by highlighting the importance of prevention and emphasizing the consequences that dental caries can have in daily life, school activities, and for the general health of the child.

Internal consistency indicates whether the items of a questionnaire measure the same phenomenon and was evaluated by the standardized Cronbach’s alpha coefficient, which assesses whether the total variance of the test results is associated with the sum of the variance from item to item, and its result may vary from –1 to +1, indicating, respectively, the maximum negative and positive correlation between the components of the measure.^22^ Values above 0.80 represent good internal consistency, but for domains with a reduced number of items, values are acceptable from 0.60.^23^ In this study, the internal consistency was ‘almost perfect’ (α>0.80) for the total IPQ–RD score and the domains “identity” and “child–consequences”; ‘substantial’ for the domains ‘parent/guardian consequences’, ‘cyclical course’, ‘disease coherence’, and ‘emotional dimensions’; and ‘moderate’ for the domains “child–control” and “parental/guardian control”. In a study conducted by Nelson *et al*.^8^ for the validation of the original version of IPQ–RD, similar results were found, mainly in the domains “identity” (α = 0.74), “consequences–children” (α =0.91), and “parent–parents consequence” (α =0.85). In a study to evaluate the psychometric properties of a version of IPQ–RD for the elderly (*Illness Perception Questionnaire Revised for Dental Use in Older/Elder Adults*, IPQ–RDE),^39^ similar results were found mainly in the domains “identity” (α = 0.81) and “consequences” (α = 0.88). These data demonstrate that the values found were similar to those of studies with the same instrument, confirming a good internal consistency.

The temporal stability of an instrument refers to the degree to which its repeated application to the same subject produces equal results, that is, it is related to the reliability of the results obtained. In this study, it was evaluated by the ICC for the total score and domains of the IPQ–RD. The ICC differentiates the variability attributable to error from actual differences in data, and the values found between 0.04 and 0.68 indicate “poor” to “substantial” test–retest reliability.^24^ Five of the 10 domains presented “poor” reproducibility: “causes of the disease” (ICC = 0.04), “emotional dimensions” (ICC = 0.24), “parent/guardian consequences” (ICC = 0.24), “child control” (ICC = 0.26), and “parental/guardian control” (ICC = 0.32). These findings can be explained by the long interval (approximately one month) between questionnaire applications. The literature suggests that test–retest reliability tends to decrease as the time between applications increses.^40^

The domains “identity” (ICC = 0.42), “disease coherence” (ICC = 0.42), and “child–consequences” (ICC = 0.45) had “moderate” test–retest reliability, and the domains “course–cyclic” (ICC = 0.64) and “chronic course” had “substantial” test–retest reliability (ICC = 0.68). In the validation study of the original instrument, reliability was tested by the calculation of weighted Kappa of 0.45 through the reapplication of the IPQ–RD in 21 participants, indicating “moderate” temporal stability.^12^ Other studies that used instruments similar to IPQ–RD did not perform the temporal stability test.^6,39,41^

This study applied the IPQ–RD, which underwent a careful protocol of translation and cultural adaptation widely used in the literature,^10,11^ensuring the understanding of the instrument by the study population, which directly affects the validation process and reliability of the measure. Moreover, the other measures used proved to be valid and reliable in previous studies, reflecting the good psychometric properties of the instruments. The limitation of the research is the sample size, which was limited due to the sanitary restrictions of the COVID–19 pandemic, and further studies with a representative sample of the population and oral evaluation of the children are needed to complement the present findings. Similarly, the test–retest reliability should be redone with a 14–day interval to avoid recall bias while ensuring that the health condition does not change to the point of affecting the data.

## Conclusions

The Brazilian version of the IPQ–RD proved to be valid and reliable to assess the cognitive and emotional perceptions of parents/guardians about caries disease in childhood.
